# Loss of Flocculus Purkinje Cell Firing Precision Leads to Impaired Gaze Stabilization in a Mouse Model of Spinocerebellar Ataxia Type 6 (SCA6)

**DOI:** 10.3390/cells11172739

**Published:** 2022-09-02

**Authors:** Hui Ho Vanessa Chang, Anna A. Cook, Alanna J. Watt, Kathleen E. Cullen

**Affiliations:** 1 Department of Physiology, McGill University, Montreal, QC H3G 1Y6, Canada; 2 Department of Biology, McGill University, Montreal, QC H3G 0B1, Canada; 3 Department of Biomedical Engineering, Johns Hopkins University, Baltimore, MD 21205, USA; 4 Department of Otolaryngology-Head and Neck Surgery, School of Medicine, Johns Hopkins University, Baltimore, MD 21205, USA; 5 Department of Neuroscience, School of Medicine, Johns Hopkins University, Baltimore, MD 21205, USA; 6 Kavli Neuroscience Discovery Institute, Johns Hopkins University, Baltimore, MD 21205, USA

**Keywords:** spinocerebellar ataxia type 6, vestibuloocular reflex, motor learning, eye movements, optokinetic reflex, saccades

## Abstract

Spinocerebellar Ataxia Type 6 (SCA6) is a mid-life onset neurodegenerative disease characterized by progressive ataxia, dysarthria, and eye movement impairment. This autosomal dominant disease is caused by the expansion of a CAG repeat tract in the *CACNA1A* gene that encodes the α1A subunit of the P/Q type voltage-gated Ca^2+^ channel. Mouse models of SCA6 demonstrate impaired locomotive function and reduced firing precision of cerebellar Purkinje in the anterior vermis. Here, to further assess deficits in other cerebellar-dependent behaviors, we characterized the oculomotor phenotype of a knock-in mouse model with hyper-expanded polyQ repeats (SCA6^84Q^). We found a reduction in the efficacy of the vestibulo-ocular reflex (VOR) and optokinetic reflex (OKR) in SCA6 mutant mice, without a change in phase, compared to their litter-matched controls. Additionally, VOR motor learning was significantly impaired in SCA6^84Q^ mice. Given that the floccular lobe of the cerebellum plays a vital role in the generation of OKR and VOR calibration and motor learning, we investigated the firing behavior and morphology of floccular cerebellar Purkinje cells. Overall, we found a reduction in the firing precision of floccular lobe Purkinje cells but no morphological difference between SCA6^84Q^ and wild-type mice. Taken together, our findings establish that gaze stabilization and motor learning are impaired in SCA6^84Q^ mice and suggest that altered cerebellar output contributes to these deficits.

## 1. Introduction

Spinocerebellar ataxias (SCAs) are a group of autosomal-dominant neurodegenerative disorders. SCAs display a highly heterogenous genotype-phenotype spectrum, but the unifying clinical symptoms observed across all SCAs include the loss of balance, gait ataxia, and slurred speech (reviewed in [1] Klockgether et al., 2019). One subtype of SCA, SCA6, is characterized by a late-onset ataxia with a relatively slow progression rate. Prior studies have reported cerebellar atrophy and Purkinje cell loss in the cerebellar hemispheres and vermis of SCA6 patients [2,3,4,5,6,7,8,9,10] In addition to their balance and gait deficits, patients with SCA6 generally display oculomotor abnormalities such as nystagmus (reviewed in [11]).

SCA6 is categorized as a polyglutamine SCA because it results from an expansion of a CAG repeat within the gene *CACNA1A* that, in turn, produces a polyglutamine (polyQ) expansion [12]. The *CACNA1A* gene encodes the α1A subunit of P/Q-type voltage-gated calcium channels. Notably, these channels are highly expressed in the cerebellum and are required for spontaneous firing properties of Purkinje cells [13,14,15]. Several mouse models that mimic the disease-causing mutation have been developed to understand the underlying pathophysiology, with the ultimate goal of developing treatments for patients with SCA6 [16,17,18,19]. Mouse models include the CT-longQ27^pc^ mutant, in which pathological C-terminus fragments of the α1A subunit of P/Q channel are overexpressed, and the SCA6^84Q^ knock-in models characterized by hyper-expanded polyQ repeats. Both models display a progressive ataxic phenotype, which begins when the animal is 7- to 8-months-old [19,20,21]. Purkinje cell degeneration was observed prior to the onset of ataxic behavior in CT-longQ27^pc^ mice [19]. On the other hand, the phenotype precedes Purkinje cell degeneration in SCA6^84Q^ mice [20].Previous studies have recorded from Purkinje cells in the cerebellar vermis in each of these two SCA6 mouse models and found a reduction in firing precision compared to wild-type mice [19,21]. 

Importantly, studies of these two SCA6 mouse models to date have focused on quantifying and understanding their resultant ataxic phenotypes. However, the SCA6 mutation affects P/Q channels throughout the entire cerebellum, including the vestibulocerebellum [2]. Thus, we predicted that other cerebellar-dependent behaviors would also be impaired. Notably, the cerebellum plays a crucial role in optimizing eye movement control for gaze stability. In particular, the vestibulo-ocular reflex (VOR) and optokinetic reflex (OKR) generate compensatory eye movements that stabilize the visual field on the retina. The flocculus of the cerebellum modulates VOR and OKR. In addition, the flocculus is involved in oculomotor learning, for example, calibrating the amplitude (gain) and direction of the VOR. Hence, we hypothesized that VOR and OKR responses and VOR motor learning may be affected in a mouse model of SCA6. 

To test this hypothesis, we recorded eye movements in SCA6^84Q^ and wild-type mice. We first quantified VOR and OKR responses and found that the gains of both reflexes were significantly reduced in SCA6^84Q^ compared to wild-type (WT) mice. In contrast, analysis of the relationship between vestibular quick phase amplitude and peak velocity revealed no differences, indicating that the observed VOR and OKR deficits were not the result of nontarget effects at the level of the extraocular motoneurons and/or their innervations. We next evaluated VOR motor learning to determine whether SCA6^84Q^ mice also demonstrated deficits during this cerebellar-dependent task. We found that VOR motor learning was indeed significantly impaired in SCA6^84Q^ mice by ~50%. Finally, because the floccular lobe of the cerebellum plays a key role in the generation of VOR, OKR, and VOR motor learning, we examined whether the firing behavior and morphology of floccular Purkinje cells were altered in SCA6^84Q^ mice. In vivo recordings revealed altered firing precision in both anesthetized and awake SCA6^84Q^ mice, but no detectable changes in morphology were found. Taken together, our results establish that gaze stability and cerebellar-dependent VOR motor learning are impaired in SCA6^84Q^ mice. We suggest changes in the firing properties of floccular Purkinje cells contribute to these observed deficits in gaze stability.

## 2. Results

### 2.1. VOR and OKR Gains Are Reduced in SCA6^84Q^ Mice

**Figure 1:**
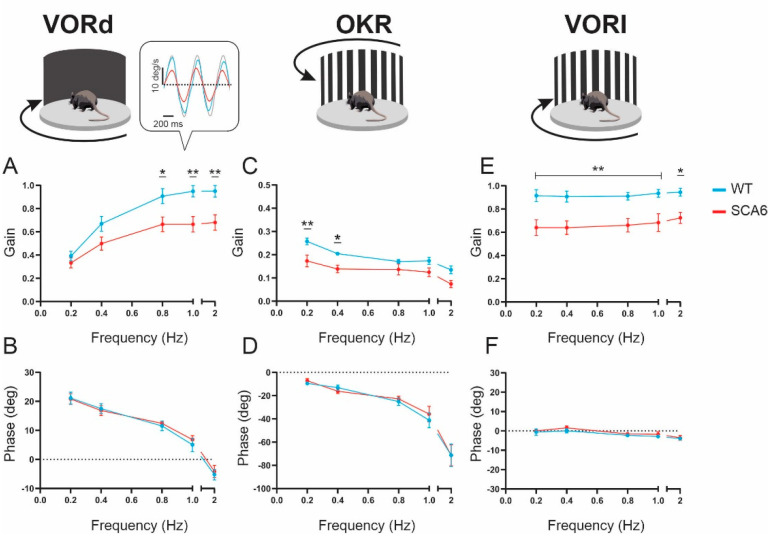
VOR and OKR gains were reduced in SCA6^84Q^ mice. (**A**,**B**) VORd gain and phase (mean ± SEM) plotted as a function of frequency for SCA6^84Q^ mice (*n* = 8) and wild-type (WT) mice (*n* = 7). VOR gains at 0.8, 1 and 2 Hz were significantly reduced in SCA6^84Q^ mice. There was no change in phase between two groups. Inset, examples of eye- and head-velocity (grey) traces for WT (blue) and SCA6^84Q^ mice (red) at 2 Hz. Note, head velocity was inverted to facilitate comparison with eye velocity. (**C**,**D**) OKR gain and phase (mean ± SEM) plotted as a function of frequency for SCA6^84Q^ mice and WT mice. OKR gains at 0.2 and 0.4 Hz were significantly reduced in SCA6^84Q^ mice with no change in phase. (**E**,**F**) VORl gain and phase (mean ± SEM) plotted as a function of frequency for SCA6^84Q^ mice and WT mice. VORl gains at all frequencies were significantly reduced in SCA6^84Q^ mice with no change in phase. Comparisons were two-way repeated measures ANOVA with post hoc *Bonferroni*’s test. * *p* < 0.05, ** *p* < 0.01.

To test whether these mice show deficits in oculomotor control, we first quantified the VOR responses of SCA6^84Q^ mice during rotation about an earth vertical axis. To measure VOR responses, head restrained mice were rotated at 0.2, 0.4, 0.8, 1, and 2 Hz with 16°/s peak velocity in the dark (referred to as VORd). We quantified the dynamic properties of VORd by computing the gain and phase for each testing frequency. Litter-matched wild-type control mice displayed robust compensatory eye movements, which increased as a function of frequency. In contrast, SCA6^84Q^ mice showed significantly reduced VOR gain at high frequencies (Figure 1A) compared to wild-type mice (wild-type vs. SCA6^84Q^ gains at 0.8 Hz, *p* = 0.021; at 1 Hz, *p* = 0.005; at 2 Hz, *p* = 0.008). At the highest frequency tested (at 2 Hz), we found a 28% decrease in VORd gain of SCA6^84Q^ mice. In contrast, there was no difference in the VOR phase between the two groups of mice (Figure 1B). We next quantified optokinetic eye movement responses (OKR) generated in response to a moving visual scene. Gains at 0.2 and 0.4 Hz were also significantly reduced in SCA6^84Q^ mice without changes in the response phase (Figure 1C,D; wild-type vs. SCA6^84Q^ gains at 0.2 Hz, *p* = 0.0046; 0.4 Hz, *p* = 0.042). Finally, VOR gain was also quantified in a lit environment (referred to as VORl), where VOR functions in concert with OKR. VOR gains of SCA6^84Q^ mice were significantly reduced at all testing frequencies with no change in the phase response (Figure 1E,F; wild-type vs. SCA6^84Q^ gains at 0.2 Hz, 0.4 Hz, 0.8 Hz, and 1 Hz, *p* < 0.01, 2 Hz, *p* = 0.021). Thus, we found that, as we hypothesized, eye movements evoked by VOR and OKR testing were altered in homozygous SCA6^84Q^ mice relative to their litter-matched controls.

### 2.2. Nystagmus Dynamics Are Comparable between Wild-Type and SCA6^84Q^ Mice

**Figure 2:**
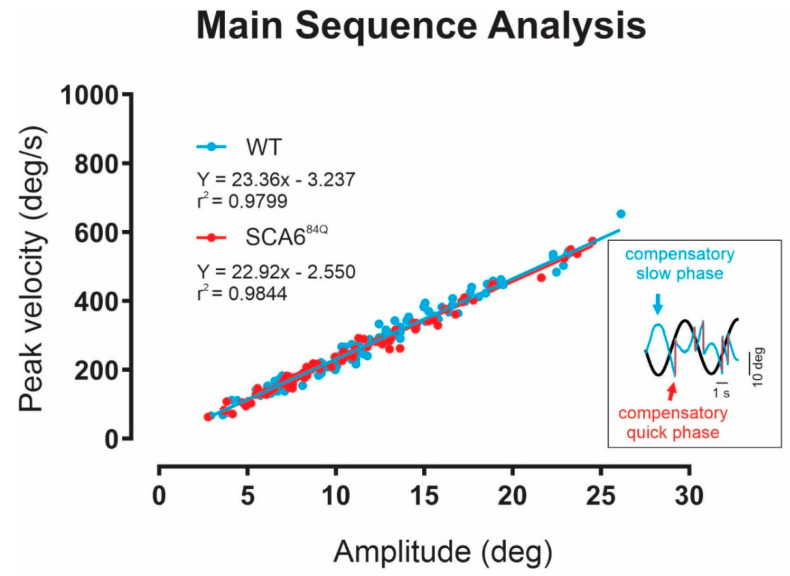
Nystagmus dynamics are comparable between wild-type and SCA6^84Q^ mice. Peak velocity vs. amplitude relationship for fast phases of vestibular nystagmus for wild-type (WT, blue dots) and SCA6^84Q^ mice (red dots). Regression lines are superimposed. There is no significant difference between the two slopes (*p* = 0.2797).

We next eliminated the possibility that the marked deficits reported above for the VOR and OKR gains of SCA6^84Q^ mice were caused by impaired processing at the level of the extraocular motoneurons and/or their innervation of extraocular muscles, rather than by impaired cerebellar function. To do this, we examined the vestibular quick phases generated by SCA6^84Q^ versus control mice (Figure 2, inset). Notably, head rotations evoke vestibular quick phases in addition to the compensatory VOR (i.e., slow phase) that we analyzed above (Figure 2, inset). Furthermore, these vestibular quick phases are known to be produced by a different premotor saccadic circuit than that which produces the compensatory VOR (i.e., slow phase) (for review see [22]). Overall, we found that quick phase amplitude was proportional to peak velocities in wild type control mice (Figure 2, blue symbols) consistent with previous studies [23,24]. Further, this relationship (commonly termed the ‘main sequence’) was comparable for SCA6^84Q^ mice (Figure 2, red symbols; *p* = 0.28). Thus, the observed deficits in the VOR and OKR gains were not due to non-target effects on extraocular motoneurons and/or their innervation of extraocular muscles. Interestingly, this result is further consistent with prior reports of normal saccade velocities in reported in SCA6 patients [25].

### 2.3. VOR Learning Is Impaired in SCA6^84Q^ Mice

**Figure 3:**
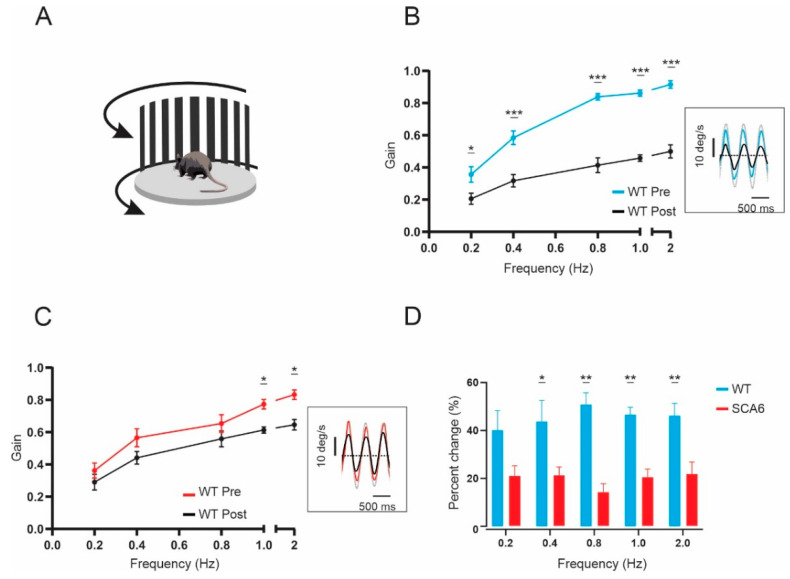
VOR learning is impaired in SCA6^84Q^ mice. (**A**) Schematic of the experimental setup. (**B**) VORd gain (mean ± SEM) before and after the VOR gain-down training plotted as a function of frequency for wild-type (WT) mice (*n* = 6). Inset, example of eye- and head-velocity (grey) traces before (blue) and after training (black) at 2 Hz (**C**) VORd gain (mean ± SEM) before and after the VOR gain-down training plotted as a function of frequency for SCA6^84Q^ mice (*n* = 7). Inset, example of eye- and head-velocity (grey) traces before (red) and after training (black) at 2 Hz (**D**) Percent change in VOR gain for WT and SCA6^84Q^ mice. Comparisons were made with two-way repeated measures ANOVA with post hoc Bonferroni’s test. * *p* < 0.05, ** *p* < 0.01, *** *p* < 0.001.

The floccular region of the cerebellum is known to play a crucial role in calibrating VOR responses. In SCA6 patients, most of the cerebellum is affected, including the floccular lobe. Thus, we hypothesized that the flocculus of the SCA6^84Q^ mice would also be affected by the mutation, and robust VOR adaptive changes would be affected. To test if SCA6^84Q^ mice show a VOR learning deficit, we carried out a 30-min-long VOR gain-down training with both wild-type and SCA6^84Q^ mice (Figure 3A). Head restrained mice were placed in the turntable that rotates together with the visual field, so the target VOR gain is 0. The training stimulus was 2 Hz with a peak velocity of 16 ◦/s. The learning acquired was assessed by quantifying change in the VOR gain after training. At the testing frequency where we expected the most significant gain decrease (2 Hz), there was an average of 46.2 ± 5.0% decrease in VOR gain after training (Figure 3B,C). In contrast, SCA6^84Q^ mice showed an average of 21.6 ± 5.0% decrease in VOR gain after training (Figure 3C,D; percent change difference at 0.4 Hz, *p* = 0.035; 0.8 Hz, *p* = 0.0012; 1 Hz, *p* = 0.0012; 2Hz, *p* = 0.0082), indicating that the SCA6^84Q^ mice were less able to adapt their eye movements to stabilize their gaze and thus have deficits in VOR learning, likely due to alterations in the floccular region of the cerebellum.

### 2.4. Firing Precision Is Altered in SCA6^84Q^ Mice

**Figure 4:**
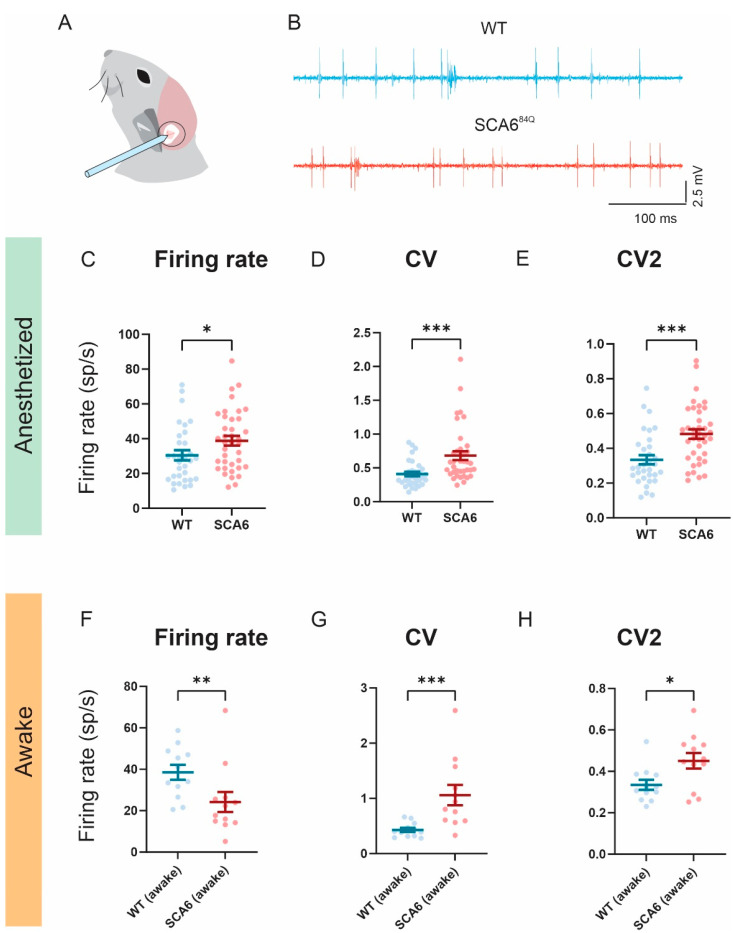
Firing precision is altered in SCA6^84Q^ mice. (**A**) Schematic of the experimental setup. (**B**) Sample recordings from anesthetized wild-type (WT, blue) and SCA6^84Q^ mice (red). (**C**–**E**) Comparison of firing rate, CV, and CV2 of simple spikes in the anesthetized state. Firing rate (**C**) is increased in SCA6^84Q^ mice (WT: frequency = 30.49 ± 2.89 sp/s; SCA6^84Q^ mice: frequency 38.83 ± 2.81 sp/s). CV (**D**) is increased in SCA6^84Q^ mice (WT:CV = 0.41 ± 0.03; SCA6^84Q^ mice: CV 0.68 ± 0.07). CV2 (**E**) is increased in SCA6^84Q^ mice (WT:CV2 = 0.33 ± 0.03; SCA6^84Q^ mice:CV2 0.48 ± 0.03). *n*= 32 cells for WT; *n* = 38 cells for SCA6^84Q^ mice (**F**–**H**) Comparison of firing rate, CV, and CV2 of simple spikes in the awake state. Firing rate (**F**) is decreased in SCA6^84Q^ mice (WT: frequency = 38.49 ± 3.58 sp/s; SCA6^84Q^ mice: frequency 24.22 ± 4.84 sp/s). CV (**G**) is increased in SCA6^84Q^ mice (WT:CV = 0.43 ± 0.04; SCA6^84Q^ mice: CV 1.06 ± 0.18). CV2 (**H**) is increased in SCA6^84Q^ mice (WT:CV2 = 0.33 ± 0.03; SCA6^84Q^ mice:CV2 0.45 ± 0.03). Comparisons were made with a non-parametric Mann–Whitney U-test. * *p* < 0.05, ** *p* < 0.01, *** *p* < 0.001.

Attenuated VOR/OKR gains and impaired VOR learning in SCA6^84Q^ mice lead us to speculate that altered neuronal firing in the flocculus may explain such deficits. Notably, a reduction in Purkinje cell firing rate and precision has been reported in the anterior vermis of SCA6^84Q^ mice, and is associated with ataxia [21]. We thus hypothesized that floccular Purkinje cells in SCA6^84Q^ mice might demonstrate comparable changes. To test this, we performed single-unit recordings in the flocculus of both anesthetized and awake mice (Figure 4A,B). We found that in the anesthetized state, Purkinje cell simple spike firing rate was significantly increased in SCA6^84Q^ mice compared to wild-type mice (*p* = 0.0218; Figure 4C). Additionally, the coefficient of variation (CV) of the inter-spike intervals (Figure 4D) in SCA6^84Q^ mice was significantly higher in SCA6^84Q^ mice, indicating reduced firing precision in SCA6^84Q^ mice (*p* = 0.0002). We also computed CV2 (see Methods) as it takes into account the presence of burst firing and pauses that are more characteristic of in vivo recordings [21,26]. This measure likewise indicated increased firing irregularity in SCA6^84Q^ mice compared to wild-type controls (*p* = 0.0002; Figure 4E). Interestingly, in awake mice, floccular Purkinje cell simple spike firing rates were significantly lower in SCA6^84Q^ mice compared to wild-type control cells (*p* = 0.0083; Figure 4F). However, both CV and CV2 values of SCA6^84Q^ mice remained higher than wild-type mice in awake mice, indicating a reduced firing precision consistent with our observations from anesthetized mice (CV, *p* = 0.004; CV2, *p* = 0.0387; Figure 4G,H). Together, these findings suggest that loss of firing precision in the floccular lobe may contribute to VOR/OKR and learning deficits in SCA6^84Q^ mice. 

### 2.5. Flocculus Morphology Appears Normal in SCA6^84Q^ Mice

**Figure 5:**
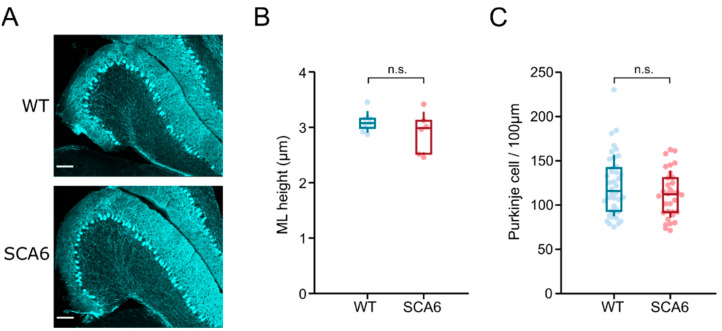
Flocculus morphology appears normal in SCA6^84Q^ mice. (**A**) Anti-calbindin immunohistochemistry in the flocculus of wild-type (WT) and SCA6 animals at 7.5 months. Anti-calbindin antibody stained Purkinje cells and showed normal flocculus morphology in SCA6^84Q^ mice. (**B**) Measurement of molecular layer height showed no change in SCA6^84Q^ flocculus, and Purkinje cell density was also unchanged (**C**). Comparisons were made with a non-parametric Mann–Whitney U-test.

Given the changes in the firing properties of Purkinje cells in the flocculus and the behavioral changes in OKR and VOR, we wondered whether the morphology of the Purkinje cells in the flocculus would be altered in SCA6. To address this possibility, we quantified the molecular layer height and Purkinje cell density. Molecular layer height is altered in other mouse models of ataxia, and in some cases is restricted to specific regions of the cerebellum (SCA1: [27]; SCA2: [28]; SCA17: [29]). Similarly, Purkinje cell loss is seen in multiple mouse models of ataxia, particularly at advanced disease stages, and can also be region specific [27,30,31,32,33]. We performed immunohistochemistry with anti-calbindin antibody to label Purkinje cells in the flocculus of litter-matched wild-type and SCA6^84Q^ mice at 7.5–8 months. This labelled Purkinje cells in the flocculus of both wild-type and SCA6^84Q^ mice (Figure 5A) and allowed visualization of the morphology of the flocculus. The flocculus appeared normal in SCA6^84Q^ mice, and analysis of both molecular layer height and Purkinje cell density showed no significant differences between wild-type and SCA6^84Q^ mice (Figure 5B,C, molecular layer height median wild-type 115.9 µm, SCA6^84Q^ 112.2 µm, *p* = 0.30; Purkinje cell density median value wild-type 3.1 cells/100 µm, SCA6^84Q^ 3.0 cells/100 µm, *p* = 0.33). Thus, Purkinje cell morphology in the flocculus appears normal at disease onset. Any differences in behavior or Purkinje cell firing appear to be arising from other changes in Purkinje cell function, similar to what we have previously observed in other regions of the cerebellum in SCA6^84Q^ mice [20,21]. 

## 3. Discussion

To date, studies of SCA6 mouse models mainly focused on deficits in motor behaviors [19,20]. Here, we show that knock-in mouse models of SCA6 with hyper-expanded polyQ repeats show a reduction in VOR and OKR responses as well as an impairment of VOR motor learning. To our knowledge, this is the first study to establish that SCA6^84Q^ mice have oculomotor deficits and impaired VOR learning. As the flocculus of the cerebellum plays a key role in the generation of VOR and OKR responses, we determined whether there were changes in the flocculus of SCA6^84Q^ mice relative to their litter-matched controls. We identified changes in the firing activity of floccular Purkinje cells in SCA6^84Q^ mice that are consistent with impaired VOR/OKR response and VOR learning. However, quantification of Purkinje cell density and molecular layer height in the flocculus showed no changes in SCA6^84Q^ mice, suggesting that Purkinje cells are not dying and have normal gross morphology at this age. Taken together, our findings suggest that mutations of the *CACNA1A* gene in SCA6^84Q^ mice lead to changes in the firing properties of floccular Purkinje cells leading to impaired gaze stabilization.

### 3.1. Comparison of Oculomotor Deficits in SCA6 Patients and a Mouse Model of SCA6

Clinical studies in human subjects have reported oculomotor deficits in SCA6 patients. While SCA6 patients generally have normal saccade velocities that fall on the main sequence, their smooth pursuit and optokinetic nystagmus gains are severely reduced [2,25]. (Reports regarding the VOR in SCA6 patients, however, show less consensus. Some studies have reported normal gains [25,34].) (While others have reported both increases [2] and decreases [35,36]) in VOR gains. Huh et al. [37] has suggested that such discrepancy can be due to difference in stimuli and severity of the disease. 

Our present experiments in SCA6^84Q^ mice similarly reveal that rapid eye movements fall on the main sequence while OKR gain is significantly reduced in SCA6^84Q^ mice. In our mouse model of SCA6, we found that VOR gain was reduced (by 28% at 2 Hz). As the cerebellum plays a vital role in visually induced motor learning, we predicted that VOR motor learning would also be impaired in SCA6^84Q^ mice. Consistent with our prediction, VOR motor learning was also significantly impaired in SCA6^84Q^ mice compared to their littermate controls (21.6% vs. 46.2% learning at 2 Hz, respectively). To date, no study to our knowledge has tested VOR learning in SCA6 patients. Nevertheless, given the cerebellum’s essential role in VOR motor learning, we speculate that these patients will show deficits similar to those reported here for SCA6^84Q^ mice. 

### 3.2. Implication of the P/Q Channel Mutation on Cerebellar-Dependent Behaviors

SCA6 is not the only disease that affects the P/Q channel alpha subunit: deletion mutations of the same gene underlie episodic ataxia type 2 (EA2) in patients, which also demonstrate gaze impairments [38].( Together these findings provide insight into the consequences of P/Q channelopathy on behavior. P/Q channels are highly expressed in the cerebellum and play a crucial role in the proper functioning of Purkinje cells. Altered P/Q channel function due to the mutation causes changes in synaptic transmission and the intrinsic properties of Purkinje cells [39,40,41]. Specifically, P/Q channels, along with calcium-activated and voltage-gated potassium channels are required for the firing precision of Purkinje cells [40,42]. As a sole output of the cerebellar cortex, we speculate that, in SCA6 mice, defective Purkinje cell signaling to downstream targets such as vestibular nuclei underlies the observed reduction in efficacy of compensatory eye movements and impaired plasticity. Indeed, mouse models with P/Q channel mutations have substantially contributed to the prevailing view of how endogenous Purkinje cell firing rhythmicity affects oculomotor phenotypes. Missense mutations in domain II and III and split-site of the *CACNA1A* gene, in which polyglutamine expansion of SCA6 mutation occurs, lead to the generation of three specific mutant mouse strains: tottering (cacna1a^tg^), rocker (cacna1a^rkr^), and leaner mice (cacna1a^tg1a^), respectively (reviewed in [43]), which have no clinical analog. Importantly, these mutant stains all demonstrate impaired gaze stabilization [44,45,46]. For example, all three of these *CACNA1A* mutant strains show reduced gains of optkinetic (OKR) and vestibulo-ocular (VOR) reflexes as well as impaired VOR learning similar to the SCA6 model mice tested in the present study [44,45,46,47].

The oculomotor impairments reported for SCA6^84Q^ mice in the present study were linked to the observed increased irregularity in floccular Purkinje cell simple spike firing (i.e., Figure 4). We have likewise previously demonstrated a relationship between ataxic behavior in SCA6^84Q^ mice and increased irregularity in anterior vermis Purkinje cell simple spike firing [21]. Further, another SCA6 mouse mutant (CT-longQ27^pc^) also displays decreased Purkinje cell simple spike firing regularity [19]. Deletion mutations of *CACNA1A* also show impairment in firing regularity in cerebellar vermis [48,49], and when firing regularity is restored, this is associated with a reduction of ataxia. Interestingly, floccular Purkinje cells of rocker and tottering mutant mice also showed increased firing irregularity scaled with the severity of the oculomotor deficit [47]. Together, these results suggest that impairments in Purkinje cell firing precision of SCA6 mice contribute to both oculomotor and motor coordination deficits.

### 3.3. Morphological Changes in the Purkinje Cells of Anterior Vermis and Flocculus in SCA6^84Q^ Mice

Histological and resonance imaging (MRI) analyses of the brains of SCA6 human patients have demonstrated the loss of Purkinje cells, and cerebellar atrophy is most prominent in the vermis of the cerebellum and, to a lesser extent, the cerebellar hemispheres (Morphology: [2,4,6]; MRI: [5,7,8,9]; Lukas et al. 2006). In this context, it is notable that our studies have identified both motor control and oculomotor dysfunctions in 7-month-old SCA6^84Q^ mice but found no significant morphological changes in Purkinje cells in either the anterior vermis or the flocculus (i.e., [20] and present study). We only observed changes in Purkinje cell density and molecular layer height at a much more advanced disease stage of 2 years of age, when Purkinje cell loss was also observed [20] Thus, our findings suggest a minimal contribution of cell loss to the impairments observed in this early disease stage in SCA6^84Q^ mice. 

It is generally believed that cerebellar cell loss follows a trend; the zebrin-positive flocculonodular lobules of cerebellum are considered to be more resistant to neurodegeneration as compared to anterior cerebellum [50]. (Additionally, in a mouse model of ARSACS, both the zebrin identity and anterior-posterior location of Purkinje cells determined their susceptibility to disease insult, both at the level of Purkinje cell loss and loss of innervation to the cerebellar nuclei [33]. Moreover, higher levels of sphingosine kinases, which are also thought to be protective against neurodegeneration, have been reported in the flocculonodular lobules compared to the anterior vermis [30]. Indeed, changes in flocculus of SCA6 patients are relatively moderate compared to the cerebellar atrophy and Purkinje cell loss reported in the vermis [2,51]. (Thus, given that several different factors appear to combine to maintain Purkinje cells in the flocculus versus vermis, we speculate that the onset of oculomotor deficits generally follows that of ataxia symptoms in SCA6 human patients. Future studies focused on the quantification of gait kinematics versus oculomotor function in patients will be required to fully understand the relative time course of SCA6 symptoms.

### 3.4. Clinical Implications

We found that the mouse model of SCA6, SCA6^84Q^ mice, displayed similar abnormal oculomotor phenotype as in human patients, highlighting the feasibility of using SCA6^84Q^ mice as the animal model for SCA6. In addition, our results contribute to the evolving idea that Purkinje cell firing deficiencies are one of the main contributing factors to motor deficits in ataxia [52]. Further studies are required to determine whether observed oculomotor deficits can be reduced by a pharmacological approach targeting the change in the intrinsic firing properties of Purkinje cells. 

## 4. Methods

### 4.1. Animals

We used a knock-in mouse model of SCA6 with a humanized 84 CAG repeat expansion mutation at the *CACNA1A* locus (SCA6^84Q^). We bred heterozygous mice (Jackson laboratories, Bar Harbor, Maine; strain: B6.129S7-Cacna1atm3Hzo/J; stock number: 008683) to obtain litter-matched SCA6^84Q^ and wild-type control mice. Genotyping was performed using primer sequences published by Jackson laboratories for this strain. All animal procedures were approved by the Johns Hopkins University and McGill University Animal Care Committees and were in accordance with the regulations established by the Canadian Council on Animal Care.

### 4.2. Quantification of VOR and OKR

The surgical procedure and experimental setup have both been previously described [53]). VOR eye movements were evoked by sinusoidally rotating the turntable at frequencies 0.2, 0.4, 0.8, 1 and 2 Hz with peak velocities of ±16°/s, in both light and dark. To evoke OKR responses, a visual cylindrical surround (vertical black and white stripes, visual angle width of 5°) was placed around the turntable and rotated at the same frequencies and velocities as mentioned above for VOR testing. An infrared video system (ETL-200, ISCAN System) was used to record eye position during VOR and OKN testing. A MEMS sensory (MPU-9250, SparkFun Electronics) was used to simultaneously record angular head velocity (the velocity of the turn table) or surround movement. Recorded eye and head movement signals were low-passed filtered at 125 Hz and sampled at 1 kHz. To compute the gain and phase of VOR and OKR compensatory eye movements, quick phases were excluded from analysis and a least-square optimization was performed (see [53]). The estimated VOR and OKR gains and phases were then plotted as mean ± standard error of the mean (SEM).

### 4.3. In Vivo Single-Unit Recording Data Acquisition and Analysis

The extracellular activity was obtained with glass micropipettes filled with 2M NaCl (Sutter Instrument). The electrode was advanced into the left flocculus using a manipulator (Narishige International, Amityville, NY, USA). Signals were amplified and band-pass filtered (400–5000 Hz). The single-unit activity was recorded using a Plexon system at a sampling rate of 20 kHz for offline analysis. Raw spike signals were imported in MATLAB (The MathWorks, Natick, MA, USA) and analyzed. The spike timing precision was quantified using a coefficient of variation (*CV*). *CV* is calculated using the formula, $CV=\frac{\sigma ISI}{\mu ISI}$, where *σ* and *μ* are the standard deviation and mean of the inter-spike intervals (*ISI*). In addition to *CV* and frequency of simple spikes, the *CV*2, which measures short-scale regularity in spike trains, was also calculated where $CV2=\frac{2\left|ISI_{n+1}-ISI_{n}\right|}{\left(ISI_{n+1}-ISI_{n}\right)}$.

### 4.4. Immunohistochemistry

Fixed tissue slices were prepared as described previously [33]. Mice were deeply anesthetized with intraperitoneal injection of 2,2,2-tribromoethanol (Avertin) prior to intracardiac perfusion with an initial flush of ice-cold phosphate-buffered saline (PBS, 0.1M, pH 7.4) with 5.6 µg/mL heparin salt. This was followed by perfusion with 40 mL of 4% paraformaldehyde (PFA) in phosphate buffer (PB, pH 7.4) before removal of the brain which was post-fixed in 4% PFA at 4 °C for 24 h. Brains were stored in PBS with 0.5% sodium azide at 4 °C until sectioning. 100 µm thick coronal slices of cerebellar tissue containing the flocculus were prepared using a Vibratome 3000 sectioning system (Concord, ON, Canada). 

We used anti-calbindin antibody to label Purkinje cells for morphological analysis. We have previously shown that this approach is a robust method for quantifying Purkinje cell degeneration and loss in another ataxia model, proving more effective than a fluorescent Nissl-like marker for carrying out Purkinje cell counts [33]. Immunohistochemistry was performed on free floating slices. Briefly, slices were incubated for 30 min in blocking solution with 5% bovine serum albumin (BSA), then washed and incubated for 3 days with anti-calbindin (1:500; CB300, Swant, Burgdorf, Switzerland) primary antibody in blocking solution followed by a 90-min incubation with AlexaFluor 488 anti-mouse (1:1000; 715-545-150, Jackson Immunoresearch, West Grove, PA, USA) secondary antibody in blocking solution. After a final wash in PBS and Triton X, slices were mounted using ProLong Gold Antifade mounting medium (ThermoFisher Scientific, Waltham, MA, USA) and stored protected from light at 4 °C.

### 4.5. Image Acquisition and Analysis

Imaging was performed using an LSM800 confocal microscope (Zeiss), using Zeiss Zen software for image acquisition. Image analysis was performed in FIJI (ImageJ; US National Institutes of Health). Molecular layer height measurements were taken at 4 points along the molecular layer and pooled. Purkinje cell density was calculated by first measuring the length of the Purkinje cell layer and then counting the number of visible Purkinje cell somas along the previously measured length.

### 4.6. Statistics

Data are reported as the mean ± SEM. Two-way repeated-measures ANOVA followed by Bonferroni post hoc comparison tests was used to test significance for the following data: (1) the VOR/OKR data across frequencies and; (2) VOR learning across frequencies. For the main sequence analysis, the extra sum-of-squares *F* test was used to compare two slopes. Non-parametric Mann–Whitney *U*-test was used to test significance for (1) percent change in VOR learning, (2) firing rate, CV, and CV2 comparison and (3) Floccular morphology comparisons. Prism 9 (GraphPad) or MATLAB was used for statistical analyses.

## Data Availability

The raw data supporting the conclusions of this article will be made available by the authors, without undue reservation.
